# mtDNAmanager: a Web-based tool for the management and quality analysis of mitochondrial DNA control-region sequences

**DOI:** 10.1186/1471-2105-9-483

**Published:** 2008-11-17

**Authors:** Hwan Young Lee, Injee Song, Eunho Ha, Sung-Bae Cho, Woo Ick Yang, Kyoung-Jin Shin

**Affiliations:** 1 Department of Forensic Medicine and Brain Korea 21 Project for Medical Science, Yonsei University College of Medicine, 250 Seongsanno, Seodaemun-gu, Seoul 120-752, Korea; 2 Department of Computer Science, Yonsei University, 262 Seongsanno, Seodaemun-gu, Seoul 120-749, Korea; 3 Department of Information and Statistics, Yonsei University, 234 Maeji-ri, Heungup-myun, Wounju-si, Gangwon-do 220-710, Korea

## Abstract

**Background:**

For the past few years, scientific controversy has surrounded the large number of errors in forensic and literature mitochondrial DNA (mtDNA) data. However, recent research has shown that using mtDNA phylogeny and referring to known mtDNA haplotypes can be useful for checking the quality of sequence data.

**Results:**

We developed a Web-based bioinformatics resource "mtDNAmanager" that offers a convenient interface supporting the management and quality analysis of mtDNA sequence data. The mtDNAmanager performs computations on mtDNA control-region sequences to estimate the most-probable mtDNA haplogroups and retrieves similar sequences from a selected database. By the phased designation of the most-probable haplogroups (both expected and estimated haplogroups), mtDNAmanager enables users to systematically detect errors whilst allowing for confirmation of the presence of clear key diagnostic mutations and accompanying mutations. The query tools of mtDNAmanager also facilitate database screening with two options of "match" and "include the queried nucleotide polymorphism". In addition, mtDNAmanager provides Web interfaces for users to manage and analyse their own data in batch mode.

**Conclusion:**

The mtDNAmanager will provide systematic routines for mtDNA sequence data management and analysis via easily accessible Web interfaces, and thus should be very useful for population, medical and forensic studies that employ mtDNA analysis. mtDNAmanager can be accessed at .

## Background

The outstanding features of human mitochondrial DNA (mtDNA) – such as its high mutation rate, absence of recombination, stability and the large number of genome copies per cell – have led to its wide utilization in various disciplines, including population, medical and forensic genetics. For the past few years, scientific controversy has surrounded the large numbers of errors detected in much of the previously published mtDNA data [1,2]. In extreme cases erroneous data can alter the main conclusion of a study [3], requiring confirmation of the absence of errors before proceeding to further analysis or drawing meaningful conclusions. Since phylogenetic investigations and database screening could have detected prevalent errors in published data sets, methodologies based on mtDNA haplogroup determination and comparisons with existing mtDNA haplotypes were proposed for preventing mtDNA errors [4,5]. In particular, the phylogenetic approach – which is the key tool used to understand the structure of the mtDNA data under study – was shown to be very useful for systematic reanalysis of an mtDNA data set. According to data and part of the phylogeny, it was reported to detect approximately 50% of all sequence errors [3] and hence has formed a starting point to localizing a sequence to a part of the phylogeny, at least to the level of the haplogroup for systematic error detection. Refinement of mtDNA phylogeny with more diagnostic mutations would facilitate the detection of more errors in mtDNA sequence data since it is based on mutation motifs, and if haplogroup determination fails, a neighbourhood search for sequences in the available database could identify a subset of potentially closely related sequences, thereby allowing researchers to pinpoint errors in the sequence by comparing the sequence in question with a limited subset of the total database [4]. However, manual haplogroup estimation requires a thorough understanding of the worldwide mtDNA phylogeny, and database screening for systematic error detection requires high-quality databases that are publicly available.

The Human Mitochondrial DataBase (HmtDB) has been designed and implemented using automatically running bioinformatics tools to facilitate mtDNA haplogroup determination [6]. The HmtDB is a database of 1255 human mitochondrial genomes annotated with population and variability data that allows researchers to analyse their own mtDNA sequences and to automatically predict their haplogroups, yielding a list of haplogroups that match. However, haplogroup determination is carried out by comparing the complete mitochondrial genome sequences with the updated mtDNA haplogroup classification based on information of the coding-region single nucleotide polymorphisms (SNPs) for about 100 mtDNA haplogroups and subhaplogroups. Accordingly, haplogroup estimation using the HmtDB would be useful for researchers dealing with complete mitochondrial genome sequences, but would not be applicable to the detection of possible errors when researchers have only mtDNA control-region sequences.

As for the database, the EDNAP (European DNA Profiling Group) mtDNA Population Database (EMPOP) is notable because it was established through a collaborative project in order to provide reliable frequency estimates for routine forensic casework [7]. The EMPOP was designed to be a high-quality, Web-based mtDNA database where primary sequence-lane data are permanently linked to compiled sequences, and phylogenetic quality control analyses are applied to data to check for errors [8]. Currently, the EMPOP contains 5173 high-quality mtDNA haplotypes that are classified into sub-Saharan African, West Eurasian, East Asian and Southeast Asian metapopulations, and thus enables users to assess the rarity of a forensic mtDNA haplotype in various populations. However, due to somewhat narrow query options and inconvenient method used to display the results, its query tool appears to be optimized for calculating frequency estimates for random matches rather than for database screening to detect possible mtDNA errors. Also, the EMPOP does not allow batch analyses. In addition to the accessibility of high-quality databases to generate reliable frequency estimates, the addition of batch analysis of mtDNA sequence data and the construction of a user's database would be greatly beneficial to forensic staff.

Here we present a Web-based bioinformatics resource called mtDNAmanager that provides a convenient interface supporting the management and quality analysis of mtDNA sequence data. The mtDNAmanager performs computations on mtDNA control-region sequences for estimating the most-probable mtDNA haplogroups, and retrieves similar sequences from a selected database. The aims of mtDNAmanager are (1) to allow researchers to automatically estimate the most-probable mtDNA haplogroups of their mtDNA control-region sequences, (2) to facilitate database screening with improved query tools and (3) to provide researchers with a convenient interface for managing and analysing their own data in batch mode. A query system in mtDNAmanager allows researchers to find sequences in the database that include queried nucleotide polymorphisms or to exhibit matches from either a selected population or the entire population. Inputted mtDNA sequences, which are either partial or whole mtDNA control-region sequences, are entered as differences relative to the revised Cambridge Reference Sequence (rCRS) [9]. During sequence searches, mtDNAmanager automatically estimates corresponding haplogroups for submitted data and calculates frequency estimates for random matches. Retrieved sequences are also annotated with the estimated haplogroup affiliation to highlight nucleotide polymorphisms that are specific to a certain group of mtDNA haplotypes. This application provides the first publicly available interface to automatically estimate the most-probable mtDNA haplogroups according to control-region mutation motifs, thereby facilitating data comparisons from a phylogenetic perspective.

## Implementation

The mtDNAmanager interface was designed to allow researchers to easily query the database and immediately view results on a single page. The mtDNAmanager Web interfaces were implemented using PHP and Asynchronous JavaScript and XML (AJAX). AJAX makes Web pages more responsive by exchanging small amounts of data with a server in the background, and thus mtDNAmanager Web pages do not have to be reloaded after each user request. This design aimed at increasing the interactivity, speed, functionality and usability of mtDNAmanager. The mtDNAmanager system is optimized for Internet Explorer version 6.0 or later.

**Figure 1:**
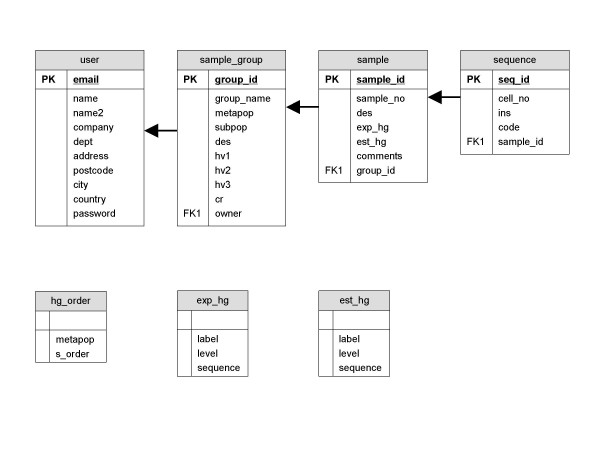
Relational database structure of mtDNAmanager.

mtDNAmanager has a multithreaded and multiuser SQL database management system designed and implemented using MySQL. The mtDNAmanager database currently comprises seven tables containing human mtDNA control-region sequences, data related to the samples and results of haplogroup estimation obtained by the mtDNA haplogroup-estimating resource that runs automatically (Figure 1).

The most-probable haplogroup of a given mtDNA sequence is estimated using a mathematical algorithm based on propositional logic via hierarchical verification of the presence or absence of haplogroup-specific diagnostic mutations. For that purpose, reliable control-region mutation motifs (strings of characteristic/diagnostic mutations shared by descent) for the assignment of more than 400 mtDNA haplogroups and subhaplogroups were first identified based on well-characterized mtDNA phylogenies (see the list of mutation motifs at ) [10-49]. Mutation motifs of most of the haplogroups could immediately be read from the mtDNA tree. However, since each position of the mutation motif displays different mutation rates and homoplasy mutations are also observed in multiple motifs, individual diagnostic positions were weighted in each haplogroup background. To this end, polymorphisms of representative haplotypes allocated to the corresponding haplogroup or subhaplogroup were screened against other closely related mtDNA haplotypes. According to the mutation stability and specificity in each haplogroup background, individual diagnostic sites were classified into clearer diagnostic mutations and their accompanying mutations. To obtain mutation frequencies, published high-quality data were mostly used, but the data found on Internet resources were also used. The clear key diagnostic mutations of a certain haplogroup could be a single mutation or a combination of multiple mutations. They were selected from the polymorphic sites observed in every haplotype of the corresponding haplogroup (100% specificity) and mostly were not shared with any other haplogroups. On the other hand, accompanying mutations are also observed in almost every haplotype of the corresponding haplogroup (>95% specificity), but could include polymorphic sites observed in another haplogroups. Based on these haplogroup-specific mutation motifs, the bioinformatics tools of mtDNAmanager designates the "expected haplogroup" when a queried data sequence possesses clear diagnostic mutations, and designates the "estimated haplogroup" when the data indicate the presence of accompanying mutations additional to the clear diagnostic mutations.

This haplogroup-estimation workflow gives priority to certain haplogroups according to their degree of specificity to corresponding population groups. Therefore, the bioinformatics tools of mtDNAmanager have a hierarchy consisting of several levels of mutation motifs. Since all of the key diagnostic mutations equally have very high specificity for their corresponding haplogroups or subhaplogroups, the levels of mutation motifs in haplogroup designation were determined by the mutation stability of each mutation motif. Therefore, within a certain haplogroup branch, subhaplogroups have a higher priority than their root haplogroups, and among haplogroups of different branches, haplogroups associated with key diagnostic sites that have a lower mutation frequency in a certain population group have a higher priority. However, since mutation frequencies and specificities differ among population groups, the order of haplogroup designations in a hierarchical analysis of diagnostic mutations varied with the population group represented in the queried sequence. In addition, for two different haplogroups with identical key diagnostic mutations, the haplogroup with the highest prevalence in a certain population group has designation priority.

The data set used to test the bioinformatics tools of mtDNAmanager contained more than 5000 mtDNA control-region sequences whose haplogroup affiliations were available from previous publications or on the Internet. Actually, the bioinformatics tools of mtDNAmanager allowed more than 98% of mtDNA to be allocated to an appropriate mtDNA haplogroup or subhaplogroup. For data sets with haplogroup information confirmed by coding-region SNPs, relatively good concordance was also observed between the expected and reference haplogroups (e.g. the concordance of 140 African Americans, 273 Austrians and 593 Koreans was 99.3%, 99.3% and 99.7%, respectively) [34,50,51].

## Results

### Content and design of the open database

The current open database of mtDNAmanager contains 7090 mtDNA control-region sequences grouped in the following five subsets: African (*n *= 1388), West Eurasian (*n *= 2857), East Asian (*n *= 1557), Oceanian and Admixed (*n *= 1288) [50-62]. All of the mtDNA control-region sequences were annotated with estimated haplogroup affiliations using the mtDNAmanager bioinformatics tools. In cases where a data sequence had been assigned to a certain haplogroup in a previous study, relevant haplogroup information is provided in the output results.

**Figure 2:**
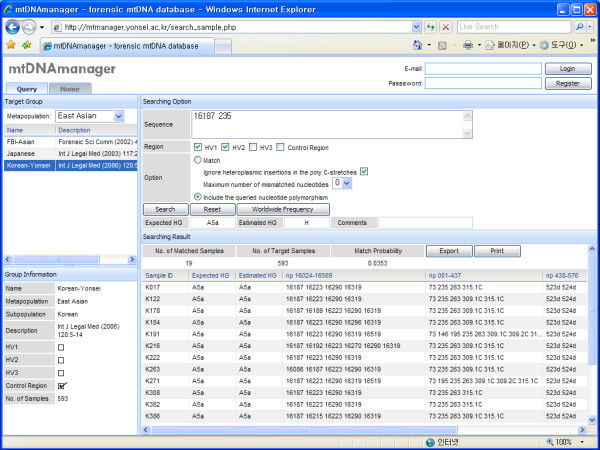
**Query page of the mtDNAmanager**. The query system – using the include setting by default – retrieves sequences that include the queried nucleotide polymorphisms from a chosen population or the entire population group of its open database. The results are displayed on the same page that the query was entered, and while displaying retrieved sequences, mtDNAmanager shows frequency estimates for random matches from a selected group and automatically estimated haplogroup affiliations for both submitted data and retrieved sequences.

The query system of mtDNAmanager retrieves sequences that include queried nucleotide polymorphisms from a selected population or the entire population group of its open database by default (exchangeable with the "include" or "match" settings). Since any combination of nucleotide polymorphisms or any partial control-region sequences can be analysed using the include setting, mtDNAmanager is very useful for analysing partial sequences or comparing similar sequences that share the same nucleotide polymorphisms (Figure 2). With the alternative setting of match, mtDNAmanager also searches sequences that match the queried sequence data from the database. mtDNAmanager provides match options to select specific regions to be analysed [HV1 (hypervariable region 1): np 16024–16365; HV2: np 73–340; HV3: np 438–576; and control-region: np 16024–16569, np 1–576], ignore heteroplasmic insertions in poly C-stretches and permit mismatches in order to overcome differences in data reporting between laboratories due to variability in the analysis region, ambiguities with respect to mtDNA nomenclature and different treatment of length variants (insertion/deletion).

The frequency of a queried nucleotide polymorphism or sequence is estimated from the number of times (*x*) that it appears in a database of size *n *(that is generally known as the counting method) while taking into account uncertainty due to sampling errors. This frequency is therefore estimated as (*x*+2)/(*n*+2) [63], and is represented as the "match probability".

### Design of user database

**Figure 3:**
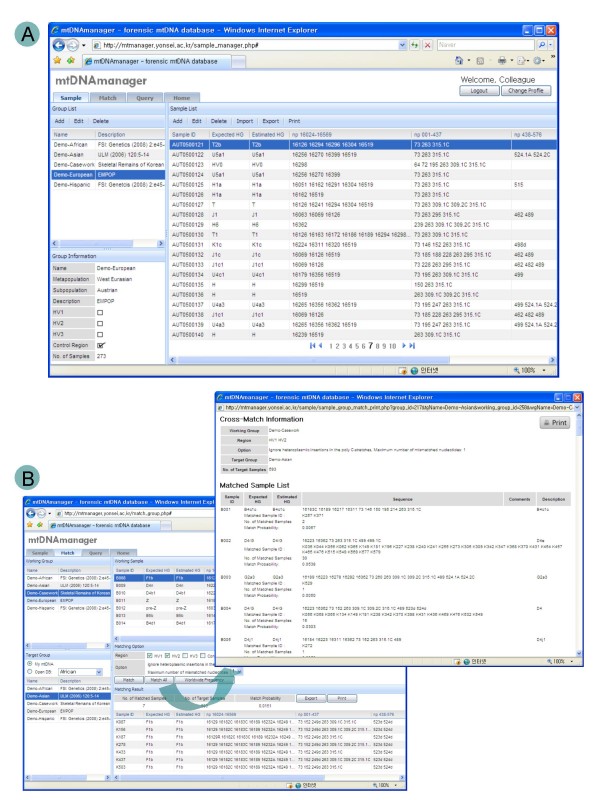
**Sample and match pages of the mtDNAmanager**. Upon registration, mtDNAmanager provides Web interfaces that allow users to submit and store their own data in batch mode and search sequences that show a match or include queried nucleotide polymorphisms from their own databases as well as mtDNAmanager's open database. (A) The sample system allows users to manage and analyse large amounts of data in batch mode. Data are characterized whilst being imported by the automatically running haplogroup-estimating workflow, and accordingly, each sample is annotated with the most-probable mtDNA haplogroup (both expected and estimated haplogroups). (B) The match system permits cross-matching of all sequence data between two selected groups as well as retrieval of matched sequences for a sample of the own database of the user or mtDNAmanager's open database. Clicking the "Match All" button will display cross-matched results in a new pop-up window.

Upon registration, mtDNAmanager provides Web interfaces through which users can submit and store their data in batch mode and search for sequences that show a match or include queried nucleotide polymorphisms from their databases as well as mtDNAmanager's open database. The sample system allows users to submit their own data in batch mode and store data in groups while simultaneously characterizing them by the automatically running haplogroup-estimating workflow (Figure 3A). The match system permits cross-matches of all sequence data between two selected groups as well as the retrieval of matched sequences for a sample of the user's database from their own database or mtDNAmanager's open database, which will facilitate casework in disasters involving many individuals (Figure 3B). In addition, the query system enables users to search sequences that show a match or include the queried nucleotide polymorphisms from their own databases or mtDNAmanager's open database, thus assisting the analysis of the increasing amount of mtDNA data available worldwide.

### Input

Input queries are entered as differences relative to the rCRS according to ISFG (International Society for Forensic Genetics) guidelines [64]. When a difference between sequence data and the rCRS is observed, only the site (which has a designated number) and nucleotide differing from the reference standard are recorded (e.g. "73G"). Insertions are recorded by first noting the site immediately 5' to the insertion followed by a decimal point and a "1" (for the first insertion), a "2" (if there is a second insertion) and so on, and then the nucleotide that is inserted is recorded (e.g. "315.1C"). Deletions are recorded by listing the missing site followed by a "d" (i.e. "249d"). For convenience, transition mutations can be recorded by listing the site and omitting the indication of the nucleotide difference. However, transversion mutations are recorded in every case (e.g. "73" versus "73C") in which the nucleotide differs from the reference standard. Polymorphic sites can be separated using a space, return or comma character. Sequence searches are allowed to show matches even when no data (i.e. no differences relative to the rCRS) have been submitted, since some Europeans possess mtDNA control-region sequences identical to the rCRS. The frequencies of nucleotide polymorphisms that are identical to the rCRS can also be obtained by entering the site and nucleotide polymorphisms of the rCRS or by entering the site with "=" (e.g. "73A" and "73=") using the include setting.

• Input sequence example 1: 16304C 73G 249d 263G 315.1C

• Input sequence example 2: 16304 73 249d 263 315.1C

To import data through the sample system in batch mode, the sample group should first be generated by the user. User-defined sample groups are added to the group list by clicking the "Add" button and entering their names and properties. Then, batch input files are prepared in a text file to be imported into a specific, user-defined group. Input files are initially prepared as Excel files that contain both the mtDNA sequence data and descriptions of the properties of the data (see examples at ). The mtDNA sequence data are entered using the same method as input queries. The Excel file is then saved as a text file (separated by tabs) that is imported to a specific user-defined group of the sample system. Input sequences can also be uploaded one by one using the "Add" button on the sample list.

### Output

Results from mtDNAmanager are displayed on the same page on which the query was submitted (Figure 2). While showing retrieved sequences, mtDNAmanager shows frequency estimates for random matches from a selected group and the automatically estimated haplogroup affiliations for submitted data. Queried nucleotide polymorphisms that are either identical to the rCRS or entered as IUPAC (International Union of Pure and Applied Chemistry) codes for point heteroplasmy are indicated as such under "Comments". Frequency estimates for all of the population groups in the database can be obtained by clicking the "Worldwide Frequency" button, and the cross-match result can be obtained by clicking the "Match All" button. The retrieved sequences are displayed with estimated haplogroup affiliations (both expected and estimated haplogroups), nucleotide polymorphisms and, if available, the haplogroup affiliations obtained from previous reports. Therefore, mtDNAmanager should facilitate the comparison of sequences that share the same nucleotide polymorphisms from a phylogenetic perspective. In addition to the Web-page presentation tools, retrieved sequences can be exported as an Excel file for user convenience.

## Discussion

The mtDNAmanager can be used to manage large amounts of mtDNA data as well as to estimate the quality of mtDNA data and compare such data with similar sequences from a phylogenetic perspective. The application provides systematic routines for error detection and strategies for screening mtDNA databases by enabling researchers to automatically estimate the most-probable mtDNA haplogroups and search the database with two alternative settings (include and match).

**Figure 4:**
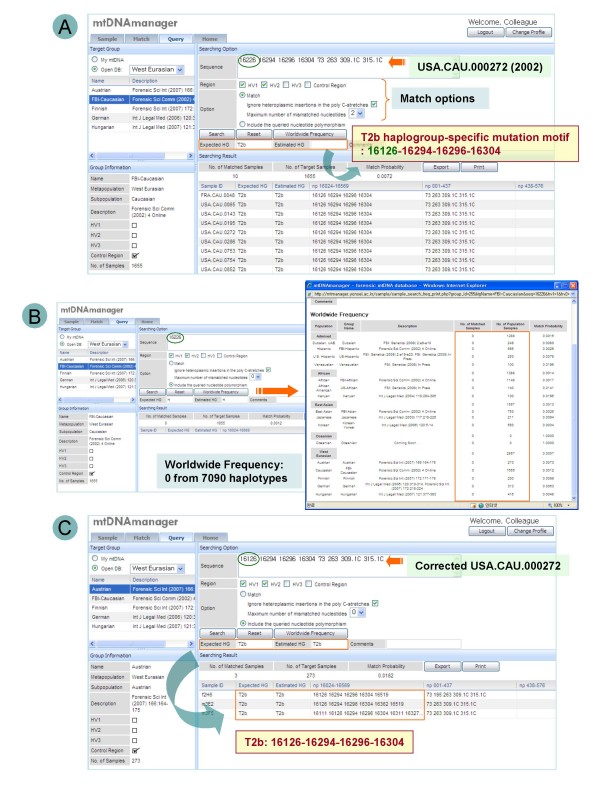
**An example of clerical error detection using mtDNAmanager**. (A) Using the match setting, USA.CAU.000272 [1,52] was analysed with the option that permits mismatch. The results showed that the sequence had no match with European populations, but the retrieved similar sequences containing mismatches all belonged to the T2b haplogroup. In addition, the original sequence was annotated with only the expected haplogroup, T2b. It was suspected that the mutation at 16226 resulted from a clerical error because the mutation motif for the T2b haplogroup suggested that the sequence lacks the 16126 mutation. (B) By clicking "Worldwide Frequency", the rarity of the 16226 mutation was investigated and the results showed that none of the 7090 mtDNA sequences bears this mutation. (C) The corrected sequence [52] was annotated with both the expected and estimated haplogroups of T2b.

**Figure 5:**
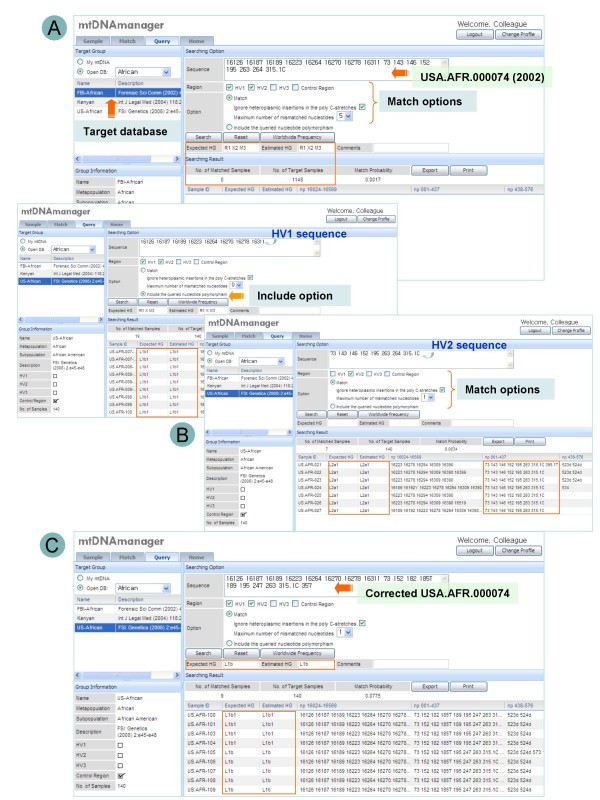
**An example of artificial recombination error detection using mtDNAmanager**. (A) USA.AFR.000074 [52,65] did not show a match with African populations or affiliations with either haplogroup. (B) Since the data set was known to be prepared from separate amplification of hypervariable regions, a possible artificial recombination was checked using query tools. The results showed that the HV1 sequence was only evident in the L1b1 haplogroup, and the HV2 sequence was only evident in the L2a1 haplogroup. (C) The corrected sequence [52] was annotated with both the expected and estimated haplogroups of L1b.

In particular, the phased designation of haplogroups (i.e. expected haplogroups and estimated haplogroups) facilitates systematic error detection by allowing the respective confirmation of the presence of clearer key diagnostic mutations and accompanying mutations. Specifically, if a certain mtDNA sequence was annotated with the same expected and estimated haplogroups, this means that the sequence possessed the complete mutation motif for the corresponding haplogroup. Likewise, if a sequence was annotated with only the expected haplogroup, this suggests a lack of accompanying mutations for the expected haplogroup, which was determined by the presence of key diagnostic mutations (Figure 4A). From an mtDNA phylogenetic perspective, this would mean that a given mtDNA haplotype is located at a previously unsampled interior node of the tree such as a back mutation [4]. Accordingly, in this case, it would be necessary to recheck the entire set of haplogroup-specific mutation sites in a given sequence data. More importantly, data without both of the haplogroup affiliations or that showed discordance between expected and estimated haplogroups would also imply a need to recheck the sequence for possible errors due to contamination or a sample mix-up during the sequencing and documentation process (Figure 5). Since mtDNA haplotypes that could only be obtained from separate amplifications of several smaller fragments – such as those found in highly degraded samples – are prone to these errors, using mtDNAmanager to confirm the absence of these errors after data generation will help to authenticate the sequence data in highly degraded samples. For user convenience, currently identified haplogroup-specific control-region mutation motifs for more than 400 haplogroups are available on the mtDNAmanager home page. From a phylogenetic perspective, mtDNA control-region sequence information might not be sufficient for assigning certain mtDNAs into respective haplogroups as reliably as the coding-region information, but the control-region mutation motifs of mtDNAmanager will at least suggest candidate sites or regions that need reinvestigation.

Frequency estimates and sequences retrieved using the include setting indicate the rarity of a nucleotide polymorphism in databases and show similar sequences that share queried nucleotide polymorphisms. Accordingly, mtDNAmanager can reveal unusual, private mutations (Figure 4B) and suggest a subset of potentially close relatives annotated with estimated haplogroup affiliations even when the haplogroup estimation of a queried sequence data fails (Figure 5B). This will highlight nucleotide polymorphisms that are specific to the retrieved group of mtDNA haplotypes and help to distinguish sites that should be analysed further. In other cases, retrieved sequences with estimated haplogroup affiliations will contribute to completing and refining haplogroup classification by revealing mutation sites that are specific to a new branch of phylogeny. Therefore, to improve mtDNA database screening, we will continue to collect and integrate high-quality mtDNA control-region sequence data that are publicly available.

In addition, mtDNAmanager provides a convenient interface that allows users to construct and analyse their own databases. Therefore, users can collect high-quality data from public databases (e.g. EMPOP) or direct sequencing results to construct their own databases. mtDNAmanager will suggest the most-probable mtDNA haplogroups for all of the sequences in the database, allowing users to also easily estimate the quality of the database. Researchers will therefore be able to select and use the most appropriate database for error detection based on their own evaluation of the quality of the available databases.

## Conclusion

The mtDNAmanager supports the management and quality analysis of mtDNA sequence data using software that performs computations on mtDNA control-region sequences for estimating the most-probable mtDNA haplogroups. mtDNAmanager will help in checking the quality of data and facilitate data comparisons from a phylogenetic perspective by displaying information – estimated haplogroup affiliations and nucleotide polymorphisms – of all sequences on a single page. In addition, mtDNAmanager provides researchers with a convenient interface for managing and analysing their own data in batch mode. Therefore, this tool could be very useful for population, medical and forensic studies that involve mtDNA analysis.

## Availability and requirements

**Project name**: A Web-based tool for the management and quality analysis of mitochondrial DNA control-region sequences

**Project home page**: 

**Operating system(s)**: Microsoft Windows

**Programming language**: PHP, Asynchronous JavaScript and XML

**Other requirements**: Optimized for Internet Explorer version 6.0 or later

**Any restrictions to use by non-academics**: None

## Authors' contributions

KJS initiated the concept of mtDNAmanager. IS and KJS developed the major modules of mtDNAmanager, and IS, HYL and KJS designed the graphical user interface. KJS reviewed and tested the software. HYL wrote this manuscript, and EH, SBC and WIY helped to draft it.
